# A database of chlorophyll *a* in Australian waters

**DOI:** 10.1038/sdata.2018.18

**Published:** 2018-02-20

**Authors:** Claire H. Davies, Penelope Ajani, Linda Armbrecht, Natalia Atkins, Mark E. Baird, Jason Beard, Pru Bonham, Michele Burford, Lesley Clementson, Peter Coad, Christine Crawford, Jocelyn Dela-Cruz, Martina A. Doblin, Steven Edgar, Ruth Eriksen, Jason D. Everett, Miles Furnas, Daniel P. Harrison, Christel Hassler, Natasha Henschke, Xavier Hoenner, Tim Ingleton, Ian Jameson, John Keesing, Sophie C. Leterme, M James McLaughlin, Margaret Miller, David Moffatt, Andrew Moss, Sasi Nayar, Nicole L. Patten, Renee Patten, Sarah A. Pausina, Roger Proctor, Eric Raes, Malcolm Robb, Peter Rothlisberg, Emily A. Saeck, Peter Scanes, Iain M. Suthers, Kerrie M. Swadling, Samantha Talbot, Peter Thompson, Paul G. Thomson, Julian Uribe-Palomino, Paul van Ruth, Anya M. Waite, Simon Wright, Anthony J. Richardson

**Affiliations:** 1 CSIRO Oceans and Atmosphere, Castray Esplanade, Hobart, TAS 7000, Australia.; 2 Climate Change Cluster (C3), University of Technology Sydney, Broadway, NSW 2007, Australia.; 3 Department of Biological Sciences, Marine Research Centre, Macquarie University, North Ryde, NSW 2109, Australia.; 4 Australian Ocean Data Network, Integrated Marine Observing System University of Tasmania, Hobart, Tasmania, 7001, Australia.; 5 Institute for Marine and Antarctic Studies, University of Tasmania, TAS 7001, Australia.; 6 Australian Rivers Institute, Griffith University, Nathan, QLD 4111, Australia.; 7 Natural Resources, Hornsby Shire Council, Hornsby NSW 2077, Australia.; 8 Waters, Wetlands and Coasts Science Branch, NSW Office of Environment and Heritage, Sydney South, NSW 1232, Australia.; 9 CSIRO Oceans and Atmosphere, EcoSciences Precinct, Dutton Park, QLD 4102, Australia.; 10 Antarctic Climate and Ecosystems Cooperative Research Centre, Hobart, TAS 7001, Australia.; 11 Evolution and Ecology Research Centre, University of New South Wales, Sydney, NSW 2052, Australia.; 12 Australian Institute of Marine Science, Townsville, QLD 4810, Australia.; 13 Sydney Institute of Marine Science, Mosman, NSW 2088, Australia.; 14 Department F.-A. Forel for Environmental and Aquatic Sciences, Earth and Environmental Sciences, University of Geneva, Geneva 4, Switzerland.; 15 Department of Earth, Ocean and Atmospheric Sciences, University of British Columbia, Columbia, Canada.; 16 CSIRO National Collections and Marine Infrastructure, Castray Esplanade, Hobart, TAS 7000, Australia.; 17 CSIRO Oceans and Atmosphere, Indian Ocean Marine Research Centre (UWA), Crawley, WA 6009, Australia.; 18 School of Biological Sciences, Flinders University, Adelaide, SA 5001, Australia.; 19 Ecosystem Health Monitoring Program, Department of Science, Information Technology and Innovation, Brisbane QLD 4001, Australia.; 20 Environmental Monitoring and Assessment Sciences, Science Division, Department of Science, Information Technology and Innovation, Brisbane QLD 4001, Australia.; 21 South Australian Research and Development Institute – Aquatic Sciences, Henley Beach, SA 5022, Australia.; 22 Environment Protection Authority, Centre for Applied Science, Ernest Jones Drive, Macleod, VIC 3085, Australia.; 23 School of Biological Sciences, The University of Queensland, St Lucia Qld 4072, Australia.; 24 School of Civil, Environmental and Mining Engineering and the UWA Oceans Institute, The University of Western Australia, Crawley, WA 6009, Australia.; 25 Alfred Wegener Institute, Helmholz Centre for Polar and Marine Research, Am Handelshafen 12, D-27570 Bremerhaven and University of Bremen, 28359 Bremen, Germany.; 26 Department of Water, Water Information and Modelling, Georges Terrace Perth, Australia.; 27 Estuary and Catchment Science, NSW Office of Environment and Heritage, Sydney South, NSW 1232, Australia.; 28 Ocean Graduate School and the UWA Oceans Institute, The University of Western Australia, Crawley, WA 6009, Australia.; 29 Southern Ocean Ecosystem Change program, Australian Antarctic Division and Antarctic Climate and Ecosystems Cooperative Research Centre, 203 Channel Hwy Kingston, Tas 7050 Australia.; 30 Centre for Applications in Natural Resource Mathematics (CARM), School of Mathematics and Physics, The University of Queensland, St Lucia, Queensland 4072, Australia.

**Keywords:** Physical oceanography, Marine biology, Marine chemistry

## Abstract

Chlorophyll *a* is the most commonly used indicator of phytoplankton biomass in the marine environment. It is relatively simple and cost effective to measure when compared to phytoplankton abundance and is thus routinely included in many surveys. Here we collate 173, 333 records of chlorophyll *a* collected since 1965 from Australian waters gathered from researchers on regular coastal monitoring surveys and ocean voyages into a single repository. This dataset includes the chlorophyll *a* values as measured from samples analysed using spectrophotometry, fluorometry and high performance liquid chromatography (HPLC). The Australian Chlorophyll *a* database is freely available through the Australian Ocean Data Network portal (https://portal.aodn.org.au/). These data can be used in isolation as an index of phytoplankton biomass or in combination with other data to provide insight into water quality, ecosystem state, and relationships with other trophic levels such as zooplankton or fish.

## Background & Summary

As the pigment chlorophyll *a* is present in all photosynthetic phytoplankton species^1^ and is relatively easy and cheap to measure, it has become a standard proxy for estimating phytoplankton biomass^2^. Samples require minimal processing and storage in the field and are not easily contaminated. Chlorophyll *a* is cheaper to process using spectrophotometry or fluorometry relative to estimating phytoplankton abundance/biomass using cell counts. Importantly, chlorophyll *a* measurements also account for the pico and nano plankton in the samples, which are substantially underestimated by phytoplankton analysts using light microscopy. These smaller size classes account for a significant fraction (commonly>70%) of total chlorophyll *a* biomass^3,4^.

However, whilst using chlorophyll *a* as an estimate of phytoplankton biomass is widespread, the relationship between the two variables is complex. Not only does the carbon to chlorophyll ratio of phytoplankton vary with species and morphological characteristics, the chlorophyll *a* content of a phytoplankton cell per unit of organic matter will vary with light intensity, nutrient availability, temperature and cell age^5–8^. Despite these complexities chlorophyll *a* remains useful as a coarse proxy for phytoplankton biomass.

In Australian waters chlorophyll *a* concentrations are generally lowest in the tropical and subtropical oceanic regions (0.05-0.5 μgL^−1^) and higher in the Southern Ocean and temperate regions (up to 1.5 μgL^−1^)^2^. In coastal zones, the chlorophyll *a* concentration can fluctuate greatly as phytoplankton blooms develop, peak and crash. The coastal station at Port Hacking, project number P782 in our database, is a good example where chlorophyll *a* concentrations typically vary between 0.1–8.0 μgL^−1^ over an annual cycle, with peaks sometimes up to 15 μgL^−1^ at 20–40 m depth coinciding with phytoplankton blooms^9^. In inshore estuaries and bays, high chlorophyll *a* values can also indicate the system is eutrophic with elevated nutrient levels from terrestrial run off. Chlorophyll *a* is therefore used in several water quality monitoring programs across the country (e.g. project number P1072 Ecosystem Health Monitoring Program in Moreton Bay, Queensland, Australia, http://healthywaterways.org/initatives/monitoring). Concentrations of chlorophyll *a* also vary throughout the oceans with oceanographic features such as upwelling and fronts which drive nutrients towards surface layers and thus enhance chlorophyll *a* levels^10,11^.

**Figure 1:**
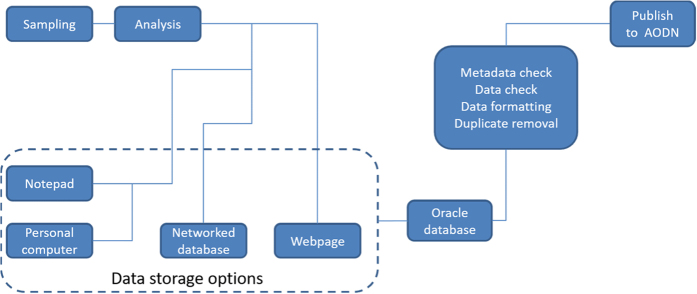
Flow diagram of data collation, verification and release to AODN.

Here we collate all available chlorophyll *a* data from Australian waters, gathered from researchers, students, government bodies, state agencies, councils and databases, along with the associated metadata through the process as detailed in Fig. 1. The chlorophyll *a* values are as measured and no attempt has been made to synthesise the data across analysis methods. The Australian Chlorophyll *a* database is available through the Australian Ocean Data Network portal (AODN: https://portal.aodn.org.au/), the main repository for marine data in Australia. The Australian Chlorophyll *a* Database will be maintained and updated through the CSIRO data centre, with periodic updates sent to the AODN. A snapshot of the Australian Chlorophyll *a* Database at the time of this publication has been assigned a DOI and will be maintained in perpetuity by the AODN (Data Citation 1).

## Methods

There are three standard methods for determining chlorophyll *a* concentrations in water samples: spectrophotometry, fluorometry and high performance liquid chromatography (HPLC). Spectrophotometric methods are described fully in Strickland and Parsons (1972)^12^, fluorometry in Zeng (2015)^13^, and HPLC in Shoaf (1978)^14^. A comprehensive discussion of the details of each method and its merits can be found in Manotura *et al.* (1997) and Roy *et al.* (2011)^15,16^.

To measure chlorophyll *a*, a known volume of water sample is filtered through a glass fibre filter paper, typically 0.45-0.7 μm pore size, under a gentle vacuum. The volume filtered varies depending on the chlorophyll *a* concentration expected in the sample, with more water filtered at lower concentrations, but the volume should be sufficient to produce a green tinge on the filter paper. Chlorophyll *a* is extracted from the filter paper with an organic solvent (e.g. acetone). Concentrations are derived from a spectrophotometer to record the light absorbance at particular wavelengths or a fluorometer that transmits an excitation beam of light in the blue range (440–460 nm) and detects the light fluoresced by chlorophyll *a* in the red wavelength range (650–700 nm). This fluorescence is directly proportional to the concentration of chlorophyll *a*. For HPLC, the filter paper is similarly extracted with an organic solvent, however pigments are then separated by passing the extract through a chromatographic column and then measured either spectrophotometrically or fluorometrically.

**Figure 2:**
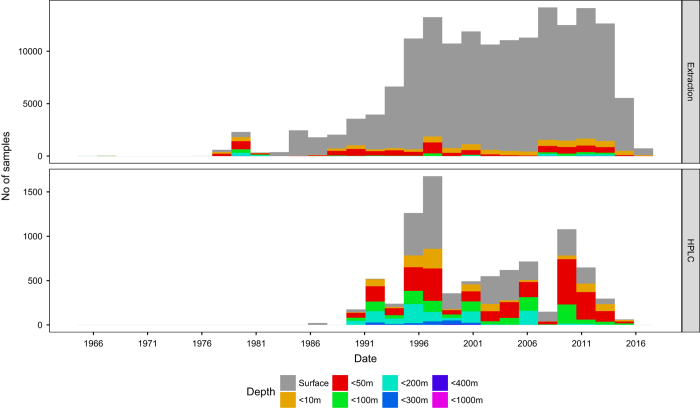
Histogram showing the range of samples over the data set period discriminated by sampling method.

Although HPLC has become the accepted benchmark for the quantification of chlorophyll *a* the volume of data collated in this database shows that spectrophotometric and fluorometric extraction methods are much more commonly used (Fig. 2). HPLC has the advantages of being more accurate and also quantifies all the other accessory pigments but it does require specialised equipment and technical skills which make it more expensive. Spectrophotometry and fluorometry are simpler and effective, but unlike HPLC they do not differentiate between chlorophyll functional types and accessory pigments. To improve the spectrophotometric and fluorometric methods, an acidifying step (e.g. addition of a small amount of hydrochloric acid) can be added after the extraction to reduce errors associated with chlorophyll degradation products^2^.

All three methods require laboratory time and sample preparation, but in-water phytoplankton biomass can also be estimated using *in-situ* fluorometers. We have excluded such observations from the current dataset because chlorophyll *a* estimates from i*n-situ* fluorometers are notoriously difficult to calibrate to an absolute standard. Although the accuracy of fluorometers is continually improving they require regular calibration, including against other methods^13^. The instruments are somewhat unstable and measurements are influenced by the presence of other environmental factors, particularly coloured dissolved organic matter (CDOM), diel, seasonal and regional effects and would also require correction for these factors^13^. The calibration routines must account for physical factors such as sensor drift, instrument design, biofouling etc. as well as the phytoplankton community composition and physiology in the sample environment, which may vary over space and time.

Data collated for this database have come from many different sources, from long-term monitoring programs run by local governments concerned with water quality to ocean voyages on research vessels. Data have been standardised to μgL^−1^, and the collection and analysis methods have been included so that inter-project comparisons can be considered. We have collated data from researchers, local and state government agencies and regional databases, e.g. AESOP (The Australian-waters Earth Observation Phytoplankton-type products) database (http://aesop.csiro.au/). The database will be maintained by the CSIRO Data Centre and updates will be available periodically through the AODN.

## Data Records

**Figure 3:**
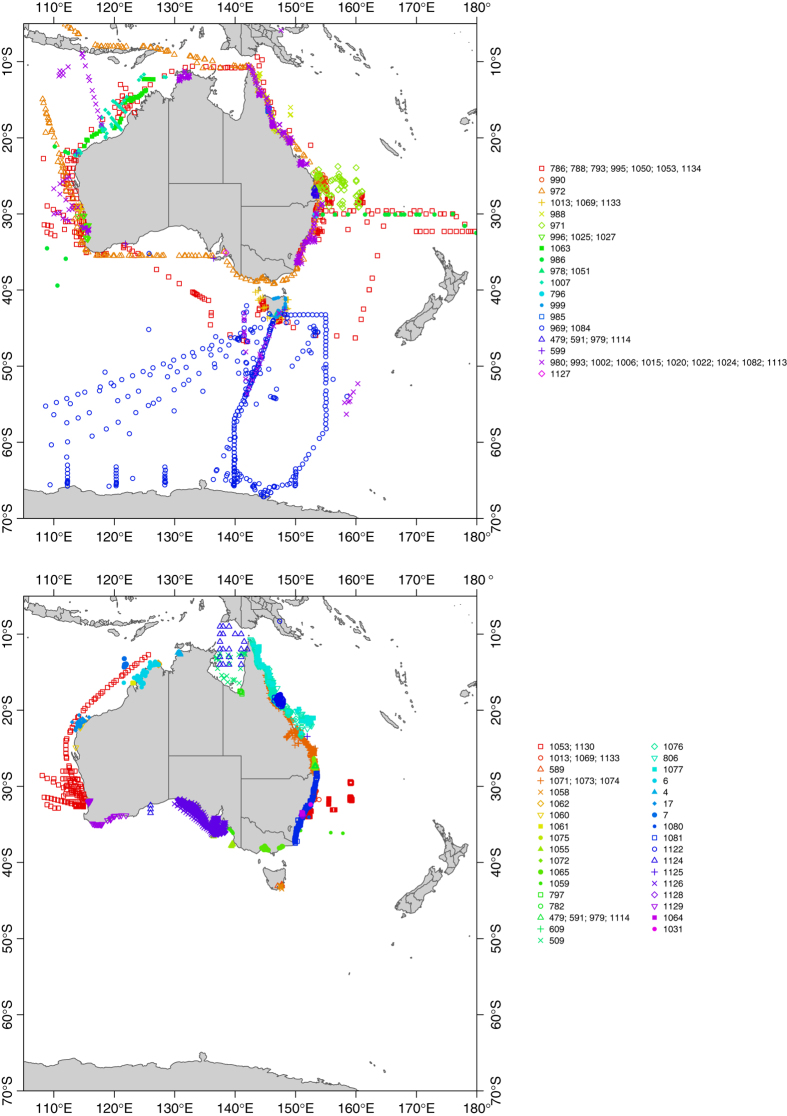
Sample locations mapped by analysis type, by HPLC and by spectrophotometry and fluorescence.

Each data record represents the chlorophyll *a* measurement taken at a point in space and time and has a unique record identification number, P(project_id)_(sample_id)_(record_id). Each data record belongs to a project, with each project having a unique identification number, Pxxx. A project is defined as a set of data records that have been collected together, usually as a cruise or study, and have the same sampling and analysis methods and the same person analysing the samples. Metadata ascribed to a project relates to all data records within that project. Details to identify each project, along with their associated samples, time and space information (Table 1 (available online only), Fig. 3) allow users to select and download discrete datasets in their area of interest. While each sample within a project has a unique sample_id, there may be more than one chlorophyll *a* record per sample if multiple replicates or depths were sampled. The sample_id has not been changed from the original data set to maintain traceability. Therefore P(project_id)_(sample_id) may be duplicated within projects, but the chlorophyll *a* records within that sample, taken at different depths for example, are given a unique record_id.

Each data record has been quality controlled. Data with insufficient or unreliable metadata were removed. All depths, times and locations have been validated and are within the boundaries expected for each project.

### Technical Validation

The database has been constructed to ensure data extraction is straight forward, although the user needs to be aware of two caveats. First, if chlorophyll *b* or other pigments are present, then fluorometry may underestimate or spectrophotometry overestimate the chlorophyll *a* concentration relative to HPLC^17–19^. When an acidification step is included, the accuracy of chlorophyll *a* from spectrophotometric and fluorometric methods is improved as effects of chlorophyll degradation products are reduced^18^. Without further pigment information comparisons between methods need to be carefully considered. This database reports values as measured and does not attempt to compare values across methods, leaving this to the discretion of the user. Second, for the HPLC data we are reporting the sum of the chlorophyll *a* pigments including the divinyl chlorophyll *a* components. The user should thus be careful when comparing data across datasets where different analysis methods have been used. Metadata have been provided in as much detail as is available so the user is aware of methodological details specific to the project.

Chlorophyll *a* values can be reported in micrograms per litre (μgL^−1^), milligrams per cubic metre (mgm^−3^) (1 μgL^−1^=1 mgm^−3^) or as depth integrated values, i.e. per square metre (mgm^−2^). In this data set we have standardised to μgL^−1^. Where depth integrated values were given, the appropriate sample depth from the study was used to convert to μgL^−1^.

All times have been converted to Coordinated Universal Time, UTC. Dates with no time component remain as reported.

The value −999 has been assigned to values that were below detection limits. The detection limit has also been included, where known, in the sample_method field of the metadata table.

### Usage Notes

This dataset and metadata has been made freely available through the AODN (Data Citation 1). The Australian Chlorophyll *a* database is complementary to the Australian Zooplankton Database^20^ and the Australian Phytoplankton Database^21^, both of which provide species-level data and are available through the AODN. Many projects in this data set have corresponding data in these species level databases and can be matched to the project via Project_id and to individual samples, via sample_id, or by using the time and date information. For example, the project 599 has data on zooplankton and phytoplankton composition, included in the aforementioned databases, plus chlorophyll *a* data in the current data set. Because the three data sets were collected at the same locations and times as part of the Integrated Marine Observing System (IMOS) National Reference Stations (NRS), they can be analysed together to investigate relationships among different trophic levels. These combined data have been used in an analysis of climate-driven variability contrasting the 2010 El Niño with the 2011 La Niña^22^. Further examples of using chlorophyll data in partnership with species-level phytoplankton and zooplankton data using data are from project 17 in the North West Cape, Western Australia^23,24^.

Projects 599, 1063, 1064, 1065, 1071, 1072, 1074, 1078 and 1129 are ongoing, and data will continue to be added to the Australian chlorophyll *a* database; for further information, contact the data custodian as listed in the metadata. The most updated version of P599 IMOS National Reference Stations, is available at: https://portal.aodn.org.au/search?uuid=f48531e2-f182-56ca-e043-08114f8c7f2e.

## Additional information

**How to cite this article**: Davies, C. H. et al. A database of chlorophyll *a* in Australian waters. *Sci. Data* 5:180018 doi: 10.1038/sdata.2017.18 (2018).

**Publisher’s note:** Springer Nature remains neutral with regard to jurisdictional claims in published maps and institutional affiliations.

## Supplementary Material



## Figures and Tables

**Table 1:** Summary of the project data included in the data set

**Project id**	**Project Name**	**Custodian**	**Acknowledgement**	**Location**	**Project start**	**Project end**	**No records**	**Methods**
1072	Estuarine Marine Ecosystem Health Monitoring Program	David Moffat (DSITI)		Moreton Bay QLD	2001-03-21	2002-02-20	50140	AeS det lim 0.1 or 0.5 μgL-1
1229	Swan River monitoring	Malcolm Robb (DOW)	DOW WA	Swan River WA	1979-03-21	2017-03-20	44569	AeS det lim 1 to 0.1 μgL-1
1073	Historical ambient chl monitoring Queensland	Andrew Moss (DSITI)		QLD	1993-09-14	2013-02-20	22690	AeS det lim 1; 2; or 0.1 μgL-1
1071	Central Queensland ambient Chl Queensland	Andrew Moss (DSITI)		Central QLD	2000-10-17	2015-03-16	13524	AeS det lim 0.1 or 0.5 μgL-1
806	CRC Reef project	Miles Furnas (AIMS)	GBRMPA	Great Barrier Reef QLD	1986-02-07	2005-01-14	6532	AeAcSF
1065	Environmental Protection Agency Victoria	Renee Patten (EPA VIC)	EPA Victoria	VIC	2008-02-21	2014-07-22	3879	AeS
1081	NSW Estuaries Chla	Peter Scanes (Env NSW)		NSW estuaries	2007-07-16	2016-04-28	3803	AeS or AeF
1122	Coastal Zone Colour Scanner	Published literature		Great Barrier Reef QLD	1979-03-06	1981-07-27	3092	AeF
1076	GBR water quality	Sam Talbot (AIMS)	AIMS	Great Barrier Reef QLD	2007-10-03	2015-07-26	3055	AeAcf
969	IDIOTS SAZ_SNARK BROKE SAZ_SENSE BROKEWEST CLIVAR KACTAS Southern Ocean Voyages^25^	Simon Wright and AADC (AAD)		Southern Ocean	1996-01-20	2007-02-19	1999	HPLC
1051	Huon Estuary Study Huon River sampling	Lesley Clementson (CSIRO)		Huon River TAS	1996-07-02	1998-10-05	1799	HPLC
1064	Hornsby Council monitoring	Peter Coad (Hornsby Council)		Hornsby NSW	1994-10-06	2015-06-23	1792	AeS det lim 0.2 μgL-1
17	North West Cape^23,24^	Sam Talbot (AIMS)		North West Cape WA	1997-10-26	2003-04-21	1603	AeAcf
1053	SS2012_T06 SS2010_V05 SS2011_V04 SS2013_V04 SS2012_V04 Southern Surveyor Voyages^26–28^	Anya Waite (UWA)		West Coast Australia	2010-07-10	2013-07-21	1202	AeAcF; HPLC
7	Scott Reef sampling^29^	Sam Talbot (AIMS)		Scott Reef WA	2008-06-24	2009-12-04	1134	AeAcf
1084	ASAC project^30–37^	Simon Wright and AADC (AAD)		Southern Ocean	1987-03-27	1997-11-10	960	HPLC
6	Kimberley sampling^38^	Sam Talbot (AIMS)		Kimberley WA	2011-01-20	2014-03-22	953	AeAcf
1126	Productivity eastern GAB	Paul van Ruth (SARDI)		GAB SA	2004-02-14	2006-03-22	838	MeS
1080	*Noctiluca scintillans*^39,40^	Jocelyn Dela-Cruz (UNSW)		Coastal NSW	1998-09-02	1999-03-11	809	AeS det lim 0.3 μgL-1
599	IMOS National Reference Stations^22^	Anthony Richardson (CSIRO)	IMOS	Australia	2009-01-15	2016-08-22	789	HPLC
797	Wet dry tropical estuary^41^	Michele Burford (Griffith University)	Tropical Rivers & Coastal Knowledge program	Gulf of Carpentaria QLD	2008-10-20	2010-06-03	551	AeAc
1077	Exchanges across the shelf-ocean boundary	Sam Talbot (AIMS)	AIMS	Great Barrier Reef QLD	2011-10-31	2014-06-18	529	AeAcf
1128	Sydney Harbour	Daniel Harrison (SIMS)		Sydney Harbour NSW	2012-10-17	2017-02-22	527	AeS
995	SS072003 SS012004 SS032006 SS092004 SS2010_V09 Southern Surveyor Voyages^42^	Lesley Clementson (CSIRO)		South West Australia	2003-08-23	2010-10-30	490	HPLC
509	Gulf of Carpentaria phytoplankton^43–45^	Michele Burford (Griffith University)	FRDC	Gulf of Carpentaria QLD	1986-04-08	2005-03-19	458	AeAcS
1031	Biogeochemical dynamics of an intermittently open estuary^46^	Jason Everett (UNSW)		Smiths Lake NSW	2002-10-03	2008-10-20	420	AeS
793	SS2010_V01 SS2011_V02 Southern Surveyor Voyages	Christel Hassler (UNIGE)		East Coast Australia	2010-01-23	2011-06-01	333	HPLC
4	Darwin Harbour	Sam Talbot (AIMS)		Darwin Harbour NT	2002-12-11	2004-12-08	320	AeAcf
1058	Storm Bay monitoring program	Kerrie Swadling (IMAS)		Storm Bay TAS	2009-11-09	2015-04-22	311	AeS
782	Port Hacking 100 m station Port Hacking 100 m station^10^	Penelope Ajani (Macquarie University)	IMOS / OEH	Port Hacking NSW	1997-04-03	2009-12-07	301	AeS
1082	Ocean Colour validation eReefs	Lesley Clementson (CSIRO)	IMOS	Great Barrier Reef QLD	1999-01-15	2014-12-15	248	HPLC
972	SOOP voyages	Lesley Clementson (CSIRO)		Australia	1999-12-01	2000-10-09	226	HPLC
589	Bloom monitoring program	Ian Jameson (CSIRO)		Australia	1993-01-14	1995-05-23	220	AeS
1025	Two Rocks SRFME - Ocean colour	Lesley Clementson (CSIRO)		Two Rocks WA	2002-02-26	2004-12-17	194	HPLC
1059	Continental Shelf	Sophie C. Leterme (Flinders University)		East Coast Australia	2011-01-28	2013-07-31	175	MeS
1074	Mackay estuaries ambient chl monitoring Queensland	Andrew Moss (DSITI)		Mackay QLD	2014-10-16	2015-02-10	144	AeS det lim 0.1 μgL-1
988	GBR_Sept2007 GBR Voyages	Lesley Clementson (CSIRO)		Great Barrier Reef QLD	2005-08-23	2008-04-23	135	HPLC
796	Phytoplankton abundance off New South Wales	Linda Armbrecht (Macquarie University)	IMOS / OEH / NSW MPA / NMSC / CSIRO	Coffs Harbour NSW	2011-05-27	2012-09-18	128	HPLC
788	SS072005 Southern Surveyor Voyages	Peter Thompson (CSIRO)		West Coast Australia	2005-07-21	2005-08-10	125	HPLC
1006	TIP2000 Ocean Colour validation	Lesley Clementson (CSIRO)		West Coast Australia	2000-09-11	2000-10-01	118	HPLC
609	Coorong Wetlands and Gulf St Vincent	Sophie C. Leterme (Flinders University)		Coorong Wetlands SA	2010-07-01	2013-08-01	110	MeS
1075	WAMSI SW Coastal Biogeography	James McLaughlin (CSIRO)	WAMSI	South Coast WA	2007-02-20	2009-12-18	109	AeS
1130	SS092006 SS102008^47,48^	Jason Everett (UNSW)		Tasman Sea	2006-09-27	2008-10-18	106	AeS
1007	North West Shelf	Lesley Clementson (CSIRO)		North West Shelf WA	1999-01-03	2003-07-17	102	HPLC
1013	FR101997 Franklin Voyages	Lesley Clementson (CSIRO)		TAS	1997-12-01	1997-12-07	99	HPLC
1134	SS2012_T03 SS2012_T07^49^	Martina Doblin (UTS)		Australia	2009-07-26	2013-08-06	99	HPLC
1027	Bunbury_2002_2005 SRFME - Ocean colour	Lesley Clementson (CSIRO)		Bunbury WA	2003-03-04	2005-02-02	92	HPLC
980	FRE_feb2008 FREsept2003 FREAug2004 FR200001_PNG FRE_feb05 Ocean Colour validation	Lesley Clementson (CSIRO)		Central QLD	2000-01-18	2008-02-09	89	HPLC
971	Sniper voyages S1 to S7	Lesley Clementson (CSIRO)		SE QLD	1999-08-16	2001-01-08	88	HPLC
1063	UWA gliders	Paul Thomson (UWA)	IMOS	WA	2015-01-18	2016-01-27	87	HPLC
1050	SS032010 Southern Surveyor Voyages	Peter Thompson (CSIRO)		NW Shelf WA	2010-04-15	2010-05-04	87	HPLC
786	SS042007	Peter Thompson (CSIRO)		West Coast WA	2007-05-16	2007-06-04	83	HPLC
1114	Moreton Bay EHMP	Emily Saeck (Griffith Uni)	Healthy Waterways	Moreton Bay QLD	2009-05-28	2011-03-03	78	HPLC
999	Tasmanian Voyages	Lesley Clementson (CSIRO)		TAS	2007-05-21	2008-12-04	78	HPLC
479	Moreton Bay floods spatial 2011	Julian Uribe Palomino (CSIRO)		Moreton Bay QLD	2011-01-19	2011-12-21	77	HPLC
990	Tonka voyages T1 to T8	Lesley Clementson (CSIRO)		SE QLD	1997-07-10	1998-11-09	75	HPLC
1133	FR14/1998^39,40^	Iain Suthers (UNSW)		East Coast Australia	1998-11-16	1998-11-20	68	AeAcS
996	JurienBay_2002_2005 SRFME - Ocean colour	Lesley Clementson (CSIRO)		Jurien Bay WA	2003-05-27	2005-02-24	65	HPLC
1060	WAMSI dredging	James McLaughlin (CSIRO)	WAMSI	Kimberley WA	2013-03-09	2015-02-26	61	AeS
1002	Laurina Voyages P1 to P4 Ocean Colour validation	Lesley Clementson (CSIRO)		NSW	1997-09-10	1998-04-27	54	HPLC
1055	Bonny and Du Coudeic Canyons	Sasi Nayar (SARDI)		Kangaroo Island SA	2008-02-07	2008-02-21	48	AeS
1062	Total Biodiversity Study	James McLaughlin (CSIRO)	Total Foundation	King George River WA	2013-06-06	2013-06-11	47	AeS
1127	Desalination project	Paul van Ruth (SARDI)		Adelaide SA	2009-06-12	2011-11-21	42	HPLC
1022	AOCWG_Apr_2003 Ocean Colour validation	Lesley Clementson (CSIRO)		West Coast Australia	2003-04-14	2003-04-17	39	HPLC
591	Moreton Bay plankton community dynamics	Sarah Pausina (UQ)	Healthy Waterways					
ARC LPO883663	Moreton Bay QLD	2009-02-04	2011-09-28	39	AeS			
985	DtreeOct2003 DtreeApr2004 Daintree samples	Lesley Clementson (CSIRO)		Daintree QLD	2003-09-29	2004-04-03	34	HPLC
1015	LB3172 Ocean Colour validation	Lesley Clementson (CSIRO)		Central QLD	2002-10-21	2002-10-29	34	HPLC
1113	North West Bay	Lesley Clementson (CSIRO)		North West Bay TAS	2006-10-02	2006-10-03	31	HPLC
1125	Roles of viruses in coral reef systems	Nicole Patten (SARDI)		Heron Island GBR QLD	2005-11-17	2005-11-27	30	AeF
1061	WAMSI Kimberley	James McLaughlin (CSIRO)	WAMSI	Kimberley WA	2014-04-01	2015-04-21	30	AeS
979	Moreton Bay Samples	Lesley Clementson (CSIRO)		Moreton Bay QLD	2003-01-28	2003-01-31	27	HPLC
1069	FR051995 Franklin Voyages	Peter Thompson (CSIRO)		West Coast Australia	1995-06-14	1995-06-15	24	HPLC
786	SS042007 Southern Surveyor Voyages	Peter Thompson (CSIRO)		West Coast Australia	2007-05-16	2007-06-04	24	HPLC
986	Beagle samples legs1;5;6	Lesley Clementson (CSIRO)		South West Australia	2003-08-04	2004-02-17	23	HPLC
993	Townsville_GBR_Jun2002 Ocean Colour validation	Lesley Clementson (CSIRO)		Townsville QLD	2002-06-05	2002-06-12	20	HPLC
1024	Elena_Rose_May_1997 Ocean Colour validation	Lesley Clementson (CSIRO)		NSW	1997-05-14	1997-05-15	11	HPLC
1020	HImay04 Ocean Colour validation	Lesley Clementson (CSIRO)		Central QLD	2004-05-19	2004-05-24	8	HPLC
Where Ae=Acetone Extraction; Me=Methanol Extraction+Ac=Acidified+S=Spectrophotometry; F=Fluorometry								

## References

[d1] Australian Ocean Data Network Davies C. H. 2017 http://dx.doi.org/10.4225/69/586f220c3f708

